# Laboratory Investigation of the Effect of Three Decontamination Methods on Surface Alterations of Dental Implants -

**DOI:** 10.31661/gmj.vi.3864

**Published:** 2025-12-16

**Authors:** Shohreh Khalilzadeh, Soroush Etesami

**Affiliations:** ^1^ Department of Prosthodontics, School of Dentistry, Ahvaz Jundishapur University of Medical Sciences, Ahvaz, Iran

**Keywords:** Dental Implants, Scanning Electron Microscope, Implant Surface Roughness

## Abstract

**Background:**

The present study aimed to evaluate the effectiveness of three decontamination methods on the surface alterations of dental implants and the removal of bacterial plaque from their surfaces.

**Materials and Methods:**

In this experimental in vitro study, 24 titanium cylinders with sandblasted, large-grit, acid-etched (SLA) surfaces were contaminated with Staphylococcus aureus to simulate biofilm formation. Samples were randomly assigned to four groups (n=6): titanium curette, diode laser, titanium brush, and saline flush control. Surface roughness (Ra and Rz) was measured using scanning electron microscopy (SEM) before and after cleaning. Colony-forming units (CFUs) were quantified post-treatment to assess bacterial removal. Statistical analyses included Kruskal-Wallis tests, Mann-Whitney pairwise comparisons, and one-way ANOVA with post hoc LSD tests (α=0.05).

**Results:**

Surface roughness differed significantly among groups after cleaning (Ra, P=.002; Rz, P=.002). Titanium curette and titanium brush produced smoother surfaces than diode laser and control, with the curette achieving the greatest reduction in roughness. CFU analysis revealed significant differences among groups (F=3.26, P=.043). Contrary to expectations, the saline flush control showed the lowest CFU counts, whereas titanium curette and titanium brush exhibited higher bacterial counts than control (P.05), and diode laser did not differ significantly from control (P=.151).

**Conclusion:**

The titanium curette and titanium brush caused the samples' most significant surface roughness changes. However, the effectiveness of these methods for bacterial plaque removal was lower than that of the control group and the Diode laser group.

## Introduction

Patients consistently seek treatment options to replace lost teeth, aiming to enhance their quality of life, functionality, and aesthetics [1]. Accordingly, tooth replacement to restore function and aesthetics has become one of the primary goals of modern dentistry [2]. Modern dental treatments are designed to restore the patient’s condition as closely as possible to a natural state, considering function, comfort, aesthetics, speech, and the health of the surrounding periodontal tissues [3].

In recent decades, implant therapy has become a common method for rehabilitation in edentulous individuals, with reported success rates of 88% in the maxilla and 93% in the mandible [4]. A dental implant is a titanium-based, root-like structure that replaces a lost tooth [5], and its success depends on the osseointegration process between the implant and the bone [6]. Despite generally high success rates, implant treatments can also be associated with failures and complications. The tissues surrounding the implant are continually exposed to various factors, including microbial plaque, chewing-related trauma, hygiene practices, and pressure from prosthetic components [7].

The presence of infection and inflammation in the peri-implant tissues can lead to implant therapy failure. In conditions such as peri-implant mucositis and peri-implantitis, inflammation extends to the soft tissues surrounding the implant [8], which may be accompanied by bone resorption and ultimately result in treatment failure. Specifically, peri-implantitis creates a favorable environment for bacterial growth on the implant surface, making plaque removal essential for long-term clinical success [9].

The implant surface itself modifies the molecular and cellular activity of surrounding tissues, providing a larger surface area for direct integration with the jawbone and thereby improving osseointegration [10]. However, these same surface irregularities also facilitate bacterial adhesion and colonization. If contamination and inflammation persist, the surrounding soft and hard tissues cannot function properly. Therefore, optimal maintenance of peri-implant tissues and, when necessary, promotion of new bone regeneration around the implant, requires thorough elimination of microbial factors from exposed implant surfaces. Additionally, in cases requiring resective surgeries, cleaning the implant surface is necessary to improve the condition of the soft tissue in the area [11].

Various methods and tools with differing effectiveness have been proposed to remove bacterial colonization from the implant surface and surrounding gingiva, with mechanical polishing using hand curettes and various materials being one of the most common approaches [12]. These tools are primarily made from plastic, carbon fiber, stainless steel, or titanium. Some studies have evaluated both the cleaning effectiveness of these materials and their potential to alter the implant surface, which can influence implant longevity [13]. For instance, one study reported that the use of sodium bicarbonate powder significantly increased implant surface roughness [14].

Al-Hashedi et al. [15] evaluated four cleaning methods—metal and plastic curettes, titanium brushes, and Er:YAG lasers—on implant surfaces. Their results indicated that none of the methods could completely remove bacterial biofilm; however, the titanium brush demonstrated higher cleaning efficacy than the other tools. Furthermore, none of the methods preserved the implant surface’s chemical properties. The study also found that laser treatment exhibited the most significant bactericidal effect on biofilm bacteria.

Taken together, given the wide range of available cleaning tools and methods and their differing potential for surface alteration, it is crucial to determine which approaches effectively clean the implant surface while minimizing surface modification. Therefore, the present study aimed to experimentally assess the surface changes of dental implants after applying three cleaning methods and to evaluate bacterial plaque removal from the implant surfaces following the use of these tools.

## Materials and Methods

### Study Design and Setting

This experimental in-vitro study was conducted at Jundi Shapur University of Medical Sciences, Ahvaz, Iran, in 2024 (1403 in the Persian calendar). The study aimed to evaluate the efficacy of three cleaning tools—titanium curette, diode laser, and titanium brush—for decontaminating titanium samples. Sample size was determined using G*Power software with a power of 80%, α=0.05, and effect size (f)=0.8, resulting in a total sample size of 24, with six samples per group (Faul et al., 2007).

Twenty-four titanium cylinders (diameter: 6 mm; height: 15 mm) were manufactured by Kousha Fan Pars (KFP Dental, Tehran, Iran). Each cylinder featured a moderately rough sandblasted, large-grit, acid-etched (SLA) surface, comparable to Nobel Biocare implants, with an attachment on one circular surface for connection to a holding shaft during cleaning. Surface roughness and porosity were initially assessed using a Leo 1455VP scanning electron microscope (SEM; Carl Zeiss AG, Jena, Germany) in atomic force microscopy (AFM) mode (non-contact) with a precision of >2.5 µm, measuring surface roughness parameters Ra (average height of peaks and valleys) and Rz (average height of surface irregularities) (Smith et al., 2016). Samples were immersed in 2.5% glutaraldehyde for 2 hours, dehydrated in increasing ethanol concentrations (30% to 100%, 15 minutes per concentration), dried to the critical point, and mounted in the SEM. SEM settings included a voltage of 20 kV and magnification of 10,000×. Three randomly selected 10 × 10 µm regions on each sample’s surface were analyzed for surface roughness.

### Biofilm Formation

To simulate contamination, samples were inoculated with Staphylococcus aureus (PTCC 1112) to form a biofilm. Each titanium cylinder was placed in a sterile, covered test tube (Aseman Lab, Tehran, Iran) and autoclaved at 121°C for 40 minutes (Firooz Dental, Tehran, Iran) to eliminate microbial contamination. A culture medium was prepared using Mueller-Hinton Broth (M391; HiMedia Laboratories, Maharashtra, India). Specifically, 2.1 g of broth was dissolved in 100 ml distilled water, boiled for 1 minute, and autoclaved at 121°C for 15 minutes. After cooling to room temperature, 5 ml of S. aureus-containing solution was added, shaken for 30 seconds, and 1 ml was pipetted into each test tube using a graduated pipette (Pars Peyvand, Tehran, Iran) to fully immerse the samples. Test tubes were sealed and incubated at 37°C for 48 hours (Pole Ideal Tajhiz, Tehran, Iran) to facilitate biofilm formation.

### Sample Processing and Bacterial Quantification

Post-incubation, samples were removed using sterile forceps and transferred to secondary test tubes containing 1 ml of sterile saline (Pars Peyvand, Tehran, Iran), previously autoclaved and cooled to room temperature. Tubes were shaken for 30 seconds to disperse bacteria. A 10-µl aliquot from each tube was collected using a laboratory loop and streaked onto Petri dishes containing Blood Agar Base (Condalab, Madrid, Spain). The agar was prepared by dissolving 40 g of powder in 1 L of distilled water, boiling for 1 minute, autoclaving at 121°C for 15 minutes, and pouring into Petri dishes (Aseman Lab, Tehran, Iran) to solidify. Petri dishes were incubated at 37°C for 24 hours in the dark to promote bacterial growth, and colony-forming units (CFUs) were counted (Jones et al., 2017).

### Cleaning Procedures

Samples were divided into four groups (n=6 per group): Group 1 (titanium curette), Group 2 (diode laser), Group 3 (titanium brush), and Group 4 (control, saline flush). Cleaning procedures were applied as follows:

1. Group 1 (Titanium Curette): Samples were cleaned using a titanium curette (Schwert, Baden-Württemberg, Germany) by a periodontal specialist applying fixed pressure. The curette was used tangentially with three consecutive 5-mm strokes per sample. No saline washing was performed to avoid interference with the control group (Lee et al., 2015).

2. Group 2 (Diode Laser): Samples were irradiated with a diode laser (Biolase, California, USA) at 400 µm wavelength, 3 W power, in continuous mode, at a 90° angle from 0.5-1 mm for 10 seconds (Lee et al., 2015).

3. Group 3 (Titanium Brush): Samples were cleaned using a titanium brush (Pocket, Dental Studio, Gyeonggi-do, South Korea) at a 45° angle, rotating at 600 rpm for 10 seconds. Saline flushing was omitted to avoid interference with the control group (Lee et al., 2015).

4. Group 4 (Control): Samples were flushed with saline using a syringe at constant pressure from 2 mm for 30 seconds.

### Post-cleaning Analysis

After cleaning, samples were transferred to third test tubes containing 1 ml of pre-autoclaved sterile saline. A 10-µl aliquot from each tube was streaked onto Blood Agar Petri dishes, incubated at 37°C for 24 hours, and CFUs were counted. Samples were dried using sterile gas and subjected to a second SEM scan (as described above) to measure changes in surface porosity (Ra and Rz). CFU counts and surface roughness parameters were compared across groups to assess cleaning efficacy and surface alterations (Smith et al., 2016; Jones et al., 2017).

### Statistical Analyses

Quantitative variables were reported as mean, standard deviation, minimum, and maximum, while qualitative variables were reported as frequency (percentage). The normality of quantitative variables was assessed using the Shapiro-Wilk test. The independent t-test or its non-parametric equivalent (Mann-Whitney test) was used to compare quantitative variables between two independent groups. Repeated measures analysis of variance (ANOVA) was applied to compare quantitative variables across more than two groups. A significance level of P<0.05 was considered for all tests. Data analysis was performed using SPSS software, version 26 .

## Results

**Figure-1:**
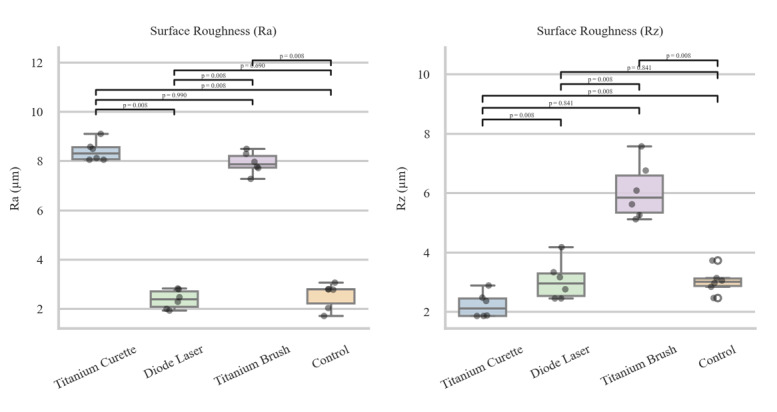
Figure 1. Mean Changes in Ra and Rz Parameters in the Samples by Type of Device Used; (P-value<0.05 considered as significant)

**Figure-2:**
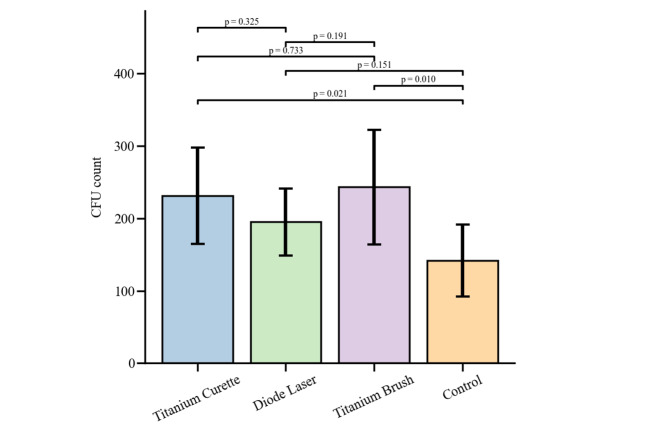
Figure 2. Mean CFU Count of the Cleaned Samples by Group

The surface roughness parameters Ra and Rz of the samples were measured using a scanning electron microscope (SEM) both before and after application of four different cleaning treatments, titanium curette (Group A), diode laser (Group B), titanium brush (Group C), and control (Group D), and the initial values were similar across all groups prior to device application. After treatment, the mean Ra values were 8.188 ± 52.74 for Group A, 2.654 ± 125.13 for Group B, 8.208 ± 110.81 for Group C, and 2.683 ± 35.63 for Group D, while the mean Rz values were 2.1279 ± 300.68 for Group A, 3.260 ± 715.96 for Group B, 6.1174 ± 512.52 for Group C, and 3.228 ± 369.14 for Group D. The Kruskal-Wallis test revealed significant differences in Ra (P=0.002) and Rz (P=0.002) across the four groups, indicating that the type of cleaning device significantly influenced surface roughness. Pairwise comparisons using the Mann-Whitney test showed that Ra and Rz were significantly different between Groups A and B (P=0.008), Groups A and D (P=0.008), Groups B and C (P=0.008), and Groups C and D (Ra P=0.008, Rz P=0.008), whereas no significant differences were observed between Groups A and C (Ra P=0.99, Rz P=0.841) or between Groups B and D (Ra P=0.69, Rz P=0.841). These results indicate that the smoothest surfaces were achieved with the titanium curette (Group A), followed by the titanium brush (Group C), whereas the highest surface roughness remained in the control group (Group D), followed by the diode laser group (Group B). Additionally, the titanium curette group exhibited the greatest reduction in surface roughness relative to the control, followed by the titanium brush group, while the diode laser group showed the least reduction in roughness compared to the control, as visually summarized in Figure-1.

Before the application of cleaning devices, the CFU (colony-forming unit) counts on all culture Petri dishes were extremely high and uncountable, indicating consistent and robust bacterial plaque formation across all samples. Therefore, pre-cleaning CFU counts were not compared, and all samples were considered to exceed 10,000 CFUs. After cleaning, the mean CFU counts differed among the groups, with Titanium Curette (231.0 ± 66.4), Diode Laser (195.0 ± 46.4), Titanium Brush (243.3 ± 79.2), and Normal Saline control (141.7 ± 49.6). One-way ANOVA revealed a statistically significant difference among the groups (F=3.26, P=0.043). Pairwise comparisons using LSD posthoc tests indicated that both Titanium Curette (P=0.021) and Titanium Brush (P=0.010) had higher CFU counts than the Normal Saline control, while Diode Laser did not differ significantly from control (P=0.151). No significant differences were observed between Titanium Brush and Titanium Curette (P=0.733), Titanium Brush and Diode Laser (P=0.191), or Titanium Curette and Diode Laser (P=0.325). Overall, the Normal Saline group showed the most effective decontamination, resulting in the lowest CFU counts, as shown in Figure-2.

## Discussion

According to the study results, the roughest surface was observed in the control group samples, followed by the samples treated with the Diode laser. Conversely, the smoothest surface was found in the samples treated with the titanium curette, followed by the titanium brush. Additionally, the smallest surface roughness change compared to the control group was observed in the Diode laser group, while the most significant surface roughness change compared to the control group was recorded in the titanium curette and titanium brush groups. Moreover, the bacterial culture results showed that using the Diode laser, similar to the control group, significantly reduced the number of bacteria remaining on the sample surfaces. In contrast, using the titanium curette and brush left a greater number of bacteria on the sample surfaces.

In a 2022 meta-analysis study by Nee et al., the effectiveness of various cleaning tools on the surface of titanium implants was examined. They concluded that using the Diode laser and LED technology for cleaning implants does not cause significant changes to their surface, which is consistent with the conclusions of our study. Additionally, this study found that using plastic curettes and rubber cup tools did not result in significant surface changes in the implants. On the other hand, the use of non-titanium metal curettes and air abrasive devices caused noticeable topographic changes to the surface of the implants. Since the other tools examined in this study were not comparable to those used in our study, their results cannot be directly compared [18] .

In a 2020 study conducted by Lollobrigida et al., the effectiveness of three tools—titanium brush, Diode laser at two power levels, and an air abrasive device—on the surface properties of titanium discs was evaluated. According to their results, surface morphological changes in the samples after using the titanium brush were significant compared to the control group, whereas the laser-treated samples did not show such changes, a conclusion that aligns with our study’s findings. Furthermore, this study revealed that none of the methods used caused significant changes in surface roughness, which also supports the findings of our study [16].

In a 2017 study conducted by Hakki et al., the effectiveness of various methods in removing debris from the surfaces of failed implants was evaluated. Similar to our study, titanium curette and titanium brush were used. The results showed that among the manual tools tested, the titanium curette had the greatest effect in removing debris from the implant surfaces, making it superior to the titanium brush. In line with this, in our study, based on the CFU count formed on the culture media after applying the tools, it can be concluded that the titanium curette was more effective than the titanium brush in eliminating bacteria, as fewer colonies were formed from the samples in that group. However, this difference was not statistically significant. Therefore, the results of our study regarding the cleaning efficiency of these tools are generally consistent with the findings reported by Hakki et al. [19].

In a 2017 study conducted by Al-Hashedi et al., the cleaning effectiveness of four different physical methods applied to titanium discs contaminated with bacterial biofilm was evaluated. Interestingly, the results showed that the number of viable bacteria on the sample surfaces after using the titanium brush was lower than on the samples cleaned with a metal curette and laser, which contrasts with our study. This discrepancy could be attributed to differences in methodology: in our study, the number of colonies formed on the culture plates after tool application was counted, whereas Al-Hashedi et al. measured the ratio of viable to dead bacteria using X-ray scanning. Additionally, the difference in results may also be related to the type of tools used, as they employed a non-titanium metal curette and an Er-YAG laser, while our study used a Diode laser and titanium curette [15].

In a 2016 study by Kushima et al., the effect of the Diode laser on the surface roughness of yttrium-stabilized zirconia samples and titanium samples with SLA surfaces was investigated. Although this study also examined temperature increases during laser application, which is not relevant to our study, it found that the surface roughness of titanium samples after laser treatment did not change significantly, a finding that is consistent with our results [20].

According to the results of our study, although the titanium curette and titanium brush created smoother surfaces in the samples, they caused the most significant surface changes compared to the control group. In contrast, the roughest surface was observed in the samples treated with the Diode laser, while these same samples exhibited the least change in surface roughness relative to the control group. The bacterial culture results also showed that the use of the Diode laser, similar to the control group, led to a significant reduction in the number of bacteria remaining on the sample surfaces. Conversely, using the titanium curette and titanium brush resulted in a higher number of bacteria remaining.

It should be noted that surface roughness changes in implants will not necessarily correlate with changes in other surface properties. For instance, evaluating alterations in the thickness of the titanium oxide layer after using cleaning tools, which plays a critical role in re-establishing osseointegration, requires additional measurement methods. Such evaluations and comparisons could be the subject of future studies. Nonetheless, the choice of cleaning method ultimately depends on the clinical goal. Some in vivo studies have proposed a Ra threshold of 0.2 μm for optimal surface roughness, suggesting that levels below this threshold can significantly reduce bacterial biofilm accumulation. In contrast, an increase in surface roughness beyond this value may promote microbial plaque accumulation, complicate oral hygiene, and create a favorable environment for periodontal disease progression around the implant [21].

Therefore, in cases involving resective treatments, implantoplasty, or other procedures where the implant surface is left exposed, methods that create smoother surfaces (such as the titanium curette and titanium brush) are likely to yield better long-term clinical outcomes. Conversely, when employing regenerative approaches that require re-osseointegration, methods like the Diode laser, which maintain a rougher surface, may be more effective.

## Conclusion

The results of the present study showed that using titanium curette and titanium brush creates a smoother surface on the samples, and the most significant surface changes in the samples compared to the control group were associated with these two tools. On the other hand, the Diode laser causes the least surface changes compared to the control group, which corresponds to the roughest surface created. Additionally, the bacterial culture results showed that using the Diode laser, like the control group, led to a significant reduction in the number of bacteria remaining on the surface of the samples. In contrast, using titanium curette and titanium brush left a higher number of bacteria on the surface of the samples.

## Conflict of Interest

None.
